# The role of dynamically induced variability in the recent warming trend slowdown over the Northern Hemisphere

**DOI:** 10.1038/srep12669

**Published:** 2015-07-30

**Authors:** Xiaodan Guan, Jianping Huang, Ruixia Guo, Pu Lin

**Affiliations:** 1 Key Laboratory for Semi-Arid Climate Change of the Ministry of Education, College of Atmospheric Sciences, Lanzhou University, Lanzhou, 730000, China; 2 Program in Atmospheric and Oceanic Sciences, Princeton University, New Jersey, 08544, USA

## Abstract

Since the slowing of the trend of increasing surface air temperature (SAT) in the late 1990 s, intense interest and debate have arisen concerning the contribution of human activities to the warming observed in previous decades. Although several explanations have been proposed for the warming-trend slowdown (WTS), none has been generally accepted. We investigate the WTS using a recently developed methodology that can successfully identify and separate the dynamically induced and radiatively forced SAT changes from raw SAT data. The dynamically induced SAT changes exhibited an obvious cooling effect relative to the warming effect of the adjusted SAT in the hiatus process. A correlation analysis suggests that the changes are dominated primarily by the North Atlantic Oscillation (NAO), Pacific Decadal Oscillation (PDO), and Atlantic Multidecadal Oscillation (AMO). Our results confirm that dynamically induced variability caused the WTS. The radiatively forced SAT changes are determined mainly by anthropogenic forcing, indicating the warming influence of greenhouse gases (GHGs), which reached levels of 400 ppm during the hiatus period. Therefore, the global SAT will not remain permanently neutral. The increased radiatively forced SAT will be amplified by increased dynamically induced SAT when the natural mode returns to a warming phase in the next period.

As a result of industrialisation, the daily mean carbon dioxide in the Earth’s atmosphere exceeded 400 parts per million (ppm) in 2013 [ http://co2now.org]. Over the past few decades, this increase had been studied due to its major global implications^1^. However, recent observations showed that the average global surface air temperature (SAT) had not risen in the 21st century, which does not fit with the simple model that directly relates warming to the increase in greenhouse gases (GHGs)^2^,^3^. The phenomenon of the WTS has been referred to as a global warming pause or hiatus, and it has attracted attention worldwide due to its apparent contradiction of the human-induced global warming theory^4^. The deviation of the observed SAT in the WTS period challenges the role of GHGs in global warming in the past century.

The cause of the WTS has been debated since its recognition by the scientific community^5^. There are two main hypotheses regarding the WTS. The first hypothesis is that the WTS is tied to natural variability: the extra heat absorbed by the climate system is not spent on warming the Earth’s surface but is instead stored in the ocean^6^,^7^. Previous results showed that natural variability plays a key role in global SAT variability^8^. Prominent decadal variabilities, such as the North Atlantic Oscillation (NAO), Pacific Decadal Oscillation (PDO), and Atlantic Multidecadal Oscillation (AMO) have been proposed as predictors or key factors in decadal simulations of the WTS. The NAO^9^ is the dominant mode of atmospheric variability over the North Atlantic region. The PDO^10^,^11^ has been identified as the cause of changes in SLP over the North Pacific, and the AMO^12^,^13^,^14^,^15^ is a measure of SST in the North Atlantic relative to the global mean.

The second hypothesis is that a reduction in the top-of-atmosphere radiative imbalance could be the result of solar variability or a stratospheric water-vapour increase^16^,^17^. The major components of the radiative forcing from increasing GHGs include carbon dioxide and several other trace GHGs, such as methane, nitrous oxide and chlorofluorocarbons, which are released into the atmosphere by human activities^18^ and impact climate change on a global scale. Other human effects, such as urban heat islands, changes in land use or land cover and irrigation, impact climate change on a regional scale^19^. Meanwhile, anthropogenic aerosols with short residency times enter the atmosphere near their sources. These tiny particulates (aerosols) in the atmosphere can cause either warming (by absorbing radiation) or cooling (by scattering and reflecting radiation back to space). In addition to anthropogenic aerosols, the radiative forcing of natural aerosols from sources such as volcanic eruptions cannot be ignored. Simple techniques have been used to remove volcanic and solar signals from SAT data. These techniques illustrate that smaller events may contribute slightly to reduced radiative forcing. However, no major volcanic eruptions have occurred since Mount Pinatubo in 1991; thus, these sources are not sufficient to slow down the global SAT warming trend^20^.

Although both hypotheses are possible, neither is generally accepted. A quantitative methodology is needed to separate the dynamical and anthropogenic SAT from the WTS. In this study, we use an advanced dynamical adjustment methodology^21^ to analyse the main cause of the WTS over the Northern Hemisphere. For the dynamical adjustment, the raw SAT is divided into two parts: one part is associated with dynamically induced forcing which is called dynamically induced SAT, the other part is associated with the build-up of GHGs and other various radiative forcings called adjusted SAT (see data and method).

## Results

**Figure 1:**
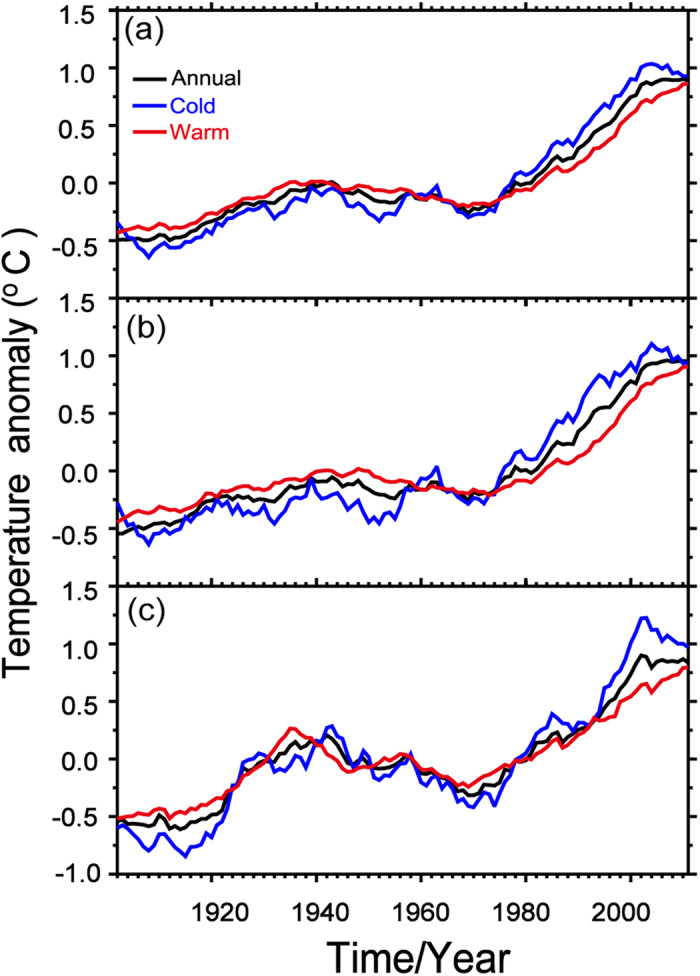
Regional mean time series of SAT anomalies based on 11-year running means from 1902 to 2011 over the Northern Hemisphere (**a**), Eurasia (**b**) and North America (**c**) for the annual (black), cold season (November to March, blue) and warm season (May to September, red). Maps and plots were made with the Interactive Data Language (IDL) software, IDL Version 8.2, License Number 1251296.

Monthly NASA Goddard Institute for Space Studies (GISS) SAT dataset^22^ is used in this study of the WTS. It is more widely in spatial cover than the other datasets as a result of involving satellite data. The annual mean SAT over Northern Hemisphere (Fig. 1a) expresses a similar curve as HadCRUT3^23^ and exhibits two hiatus periods of 1940–1970 and 2000–2011. The annual mean SAT increased continuously between the two hiatus periods from 1970 to 2000 (Fig. 1a). The mean SAT in the cold season decreased over Eurasia (Fig. 1b) and North America (Fig. 1c) and was offset by continuously increasing SAT in the warm season. We conclude that for approximately 10 years after 2000 (when the WTS began), the decreasing SAT in the cold season led to the annual mean SAT slowdown that occurred over the Northern Hemisphere (see Fig. 1). The SAT hiatus has only occurred in the cold season over the Northern Hemisphere^24^. Therefore, we confined our analysis to SAT changes in the cold season of the Northern Hemisphere.

**Figure 2:**
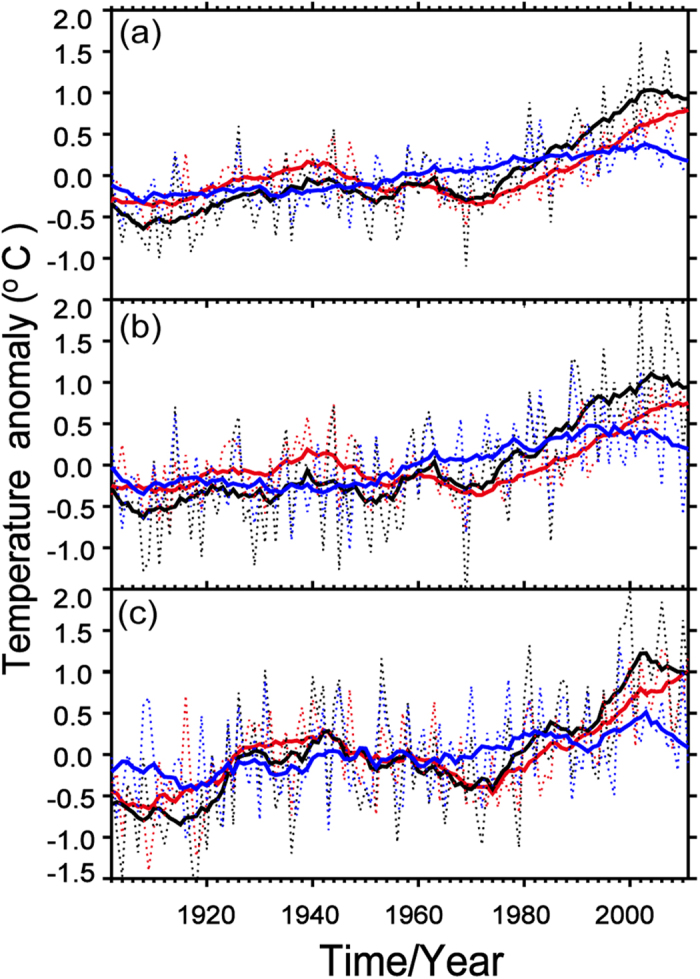
Regional mean time series of the raw (dashed black), dynamically induced (dashed blue) SAT anomalies and adjusted (dashed red) SAT anomalies with 11-year running means of raw (solid black), dynamically induced (solid blue) and adjusted (solid red) in the cold seasons (November to March) of 1902 to 2011 over the Northern Hemisphere (**a**), Eurasia (**b**) and North America (**c**). Maps and plots were made with the Interactive Data Language (IDL) software, IDL Version 8.2, License Number 1251296.

For the variability in the average SAT over the Northern Hemisphere, the raw SAT exhibits a continuous increase started from 1970 s until the onset of the hiatus in approximately 2000, followed by a steady trend (Fig. 2a). Since the 11-year running is a strong filter for removing decadal signal, its time series in Fig.1 is more like the adjusted SAT anomalies in Fig. 2. Remarkably, the adjusted SAT time series exhibits a linear increase after 1970 until 2000, follow a relative lower increasing rate of warming in the hiatus, accompany with the obvious cooling trend of dynamically induced SAT in Fig. 2a, whereas demonstrated the role of the dynamically induced variability in the WTS. The dynamically induced SAT as a part of raw SAT represents the interannual to interdecadal variability in the period of 1902–2011. When this part is in the uptrend phase (warming), it made the positive contribution to accelerating warming in the period of 1980–2000. When this part is in the downtrend phase (cooling), however, it reduced or even balanced the radiatively forced warming in the recent WTS (hiatus) period started from 2000 to now. For the regional average SAT time series in Fig. 2b,c, a significant slowdown in the raw SAT, along with an increase in the adjusted SAT, occurred over Eurasia and North America. It indicated that adjusted SAT most play a warming role in the SAT change. Although the first hiatus in the period of 1940–1970 also exhibits both dynamically and radiatively induced SAT contribution, it is different with the recently hiatus, which is a result of the balance between dynamically induced SAT and radiatively forced SAT. The radiatively forced SAT (adjusted SAT) in the first hiatus period is much weaker than it in current hiatus period and dynamically induced SAT took the dominated role in the raw SAT variation. Therefore, the dynamically and radiatively induced SAT exhibited different contribution to the raw SAT change in the two hiatus periods. The mechanism of hiatus in the period of 1940–1970 needs to be further studied.

**Figure 3:**
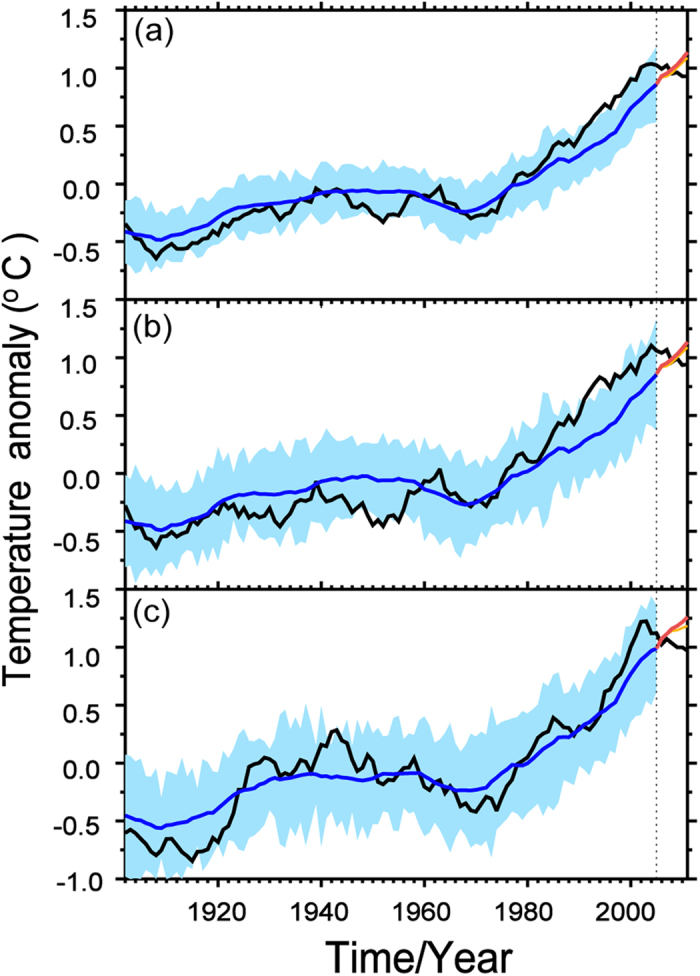
Regional mean time series of observational (black) and ensemble-mean CMIP5 simulations (blue) SAT anomalies based on 11-year running means over the Northern Hemisphere (**a**), Eurasia (**b**) and North America (**c**) in the cold season (November to March) of 1902 to 2011. The blue shading indicates the standard deviation of the CMIP5-simulated SAT. The yellow and orange curves are the regional mean SAT projections from RCP 4.5 and RCP 8.5, respectively. Maps and plots were made with the Interactive Data Language (IDL) software, IDL Version 8.2, License Number 1251296.

The adjusted SAT is always been referred to as “radiatively forced SAT” by scientist, the biggest effect comes from increasing carbon dioxide in the atmosphere because carbon dioxide is the major greenhouse gases (GHGs) that is most responsible for the SAT warming. However, it still contains methane, nitrous oxide, chlorofluorocarbons, urban heat island effect, land use and so on that increased from various human activities, while play positive and negative roles in climate change. In order to manifest the effect of GHGs in adjusted SAT, a comparison between the observed SAT and a 20-model^25^ ensemble mean of CMIP5 simulations over the Northern Hemisphere (Fig. 3a) has been plotted and showed that the time series of the CMIP5 simulations is much smoother than the observed SAT curve and that the two datasets do not agree regarding the timing of the WTS that started from the 21st century. In particular, the notable discrepancy between the observed and simulated SAT illustrates the failure of the CMIP5 models to capture the enhanced warming^26^ from 1975 to 1999 and the WTS from 2000 to 2011 during the cold season. The observed average SAT over Eurasia (Fig. 3b) shows a significant decrease during the WTS period, while a continuous SAT increase is observed in the CMIP5 models results. In addition, the observed and simulated SAT over North America (Fig. 3c) illustrate similar curves to those in Eurasia (Fig. 3b), with a warming halt and an increasing CMIP5 ensemble mean SAT, respectively. It indicated that the warming effect of climate change that induced by increasing GHGs in model dominated the simulated SAT variability. Meanwhile, The similar warming trend between CMIP5 in Fig. 3 and adjusted SAT in Fig. 2 illustrated the warming effect of radiative factors in the process of SAT variability and proved the cooling effect of dynamically induced SAT in WTS.

**Figure 4:**
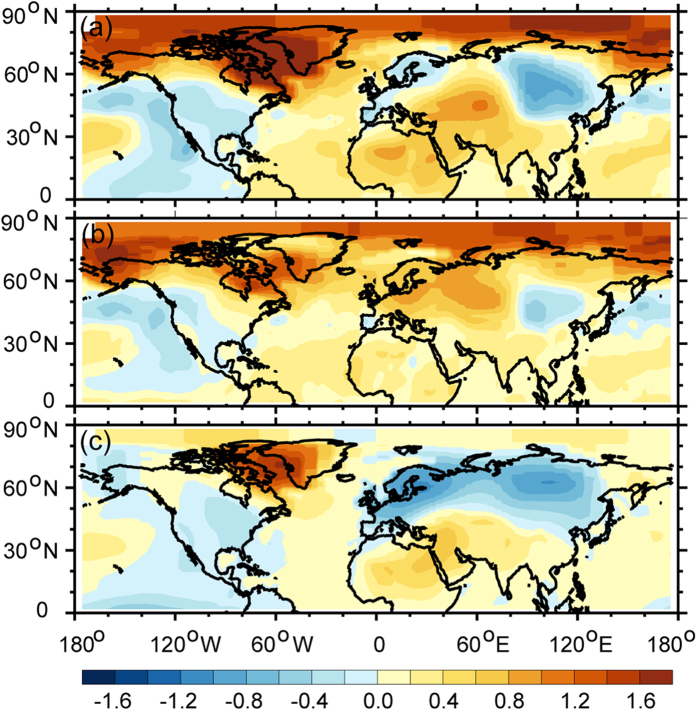
The difference between the WTS period (2001–2011) and the previous decade (1991–2000) of raw (**a**), adjusted (**b**) and dynamically induced (**c**) SAT. Maps and plots were made with the Interactive Data Language (IDL) software, IDL Version 8.2, License Number 1251296.

**Figure 5:**
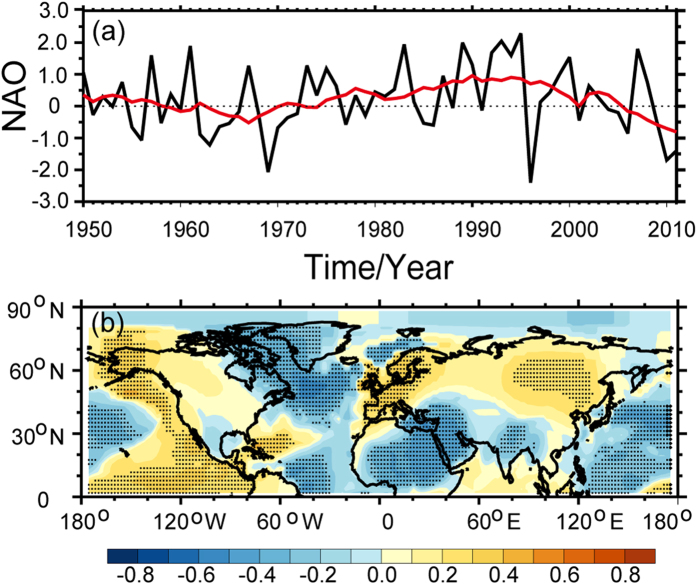
The NAO time series for the period 1950–2011 in cold season, the red line represents the 11-year running mean (**a**). Spatial distribution of the correlation coefficient between the NAO (detrended and 11-year running means) and the detrended dynamically induced SAT during the cold seasons of 1950 to 2011(**b**). The stippling indicates a 95% confidence level according to a two-tailed Student’s t-test. Maps and plots were made with the Interactive Data Language (IDL) software, IDL Version 8.2, License Number 1251296.

**Figure 6:**
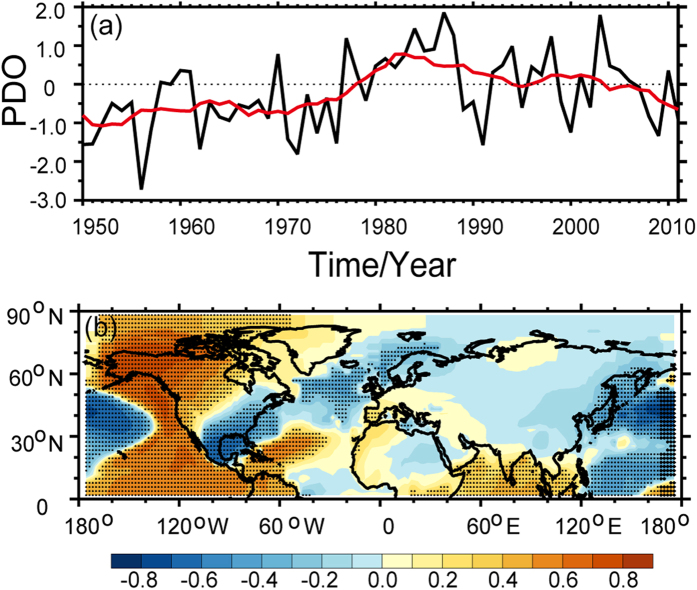
The PDO time series for the period 1950–2011 in cold season, the red line represents the 11-year running mean (**a**). Spatial distribution of the correlation coefficient between the detrended PDO and the detrended dynamically induced SAT during the cold seasons of 1950 to 2011 (**b**). The stippling indicates a 95% confidence level according to a two-tailed Student’s t-test. Maps and plots were made with the Interactive Data Language (IDL) software, IDL Version 8.2, License Number 1251296.

**Figure 7:**
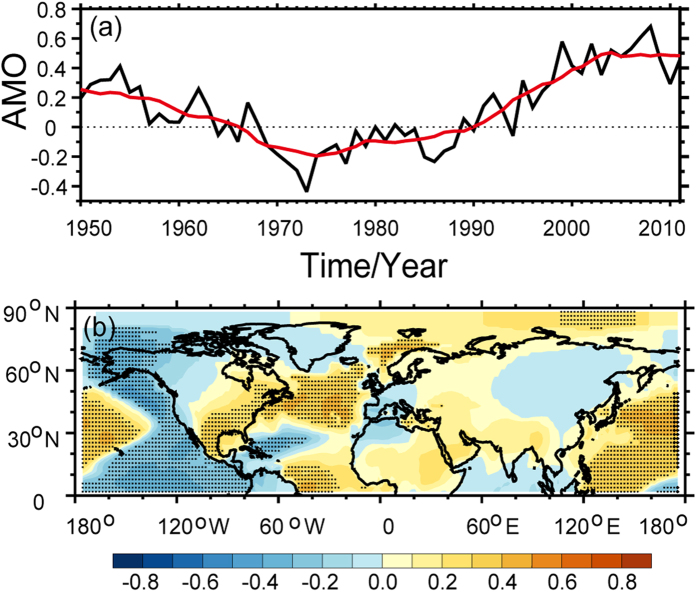
Same as in Fig. 6, but for AMO. Maps and plots were made with the Interactive Data Language (IDL) software, IDL Version 8.2, License Number 1251296.

As shown in the time series of observed SAT in Fig. 3, it must happen cooling events when it turned from enhanced warming into the WTS. The obvious difference between previous decade and WTS period will illustrate the scale of warming and cooling in instrumental records^27^. In order to explore the scale distribution, we plotted the epochal difference of the GISS SAT dataset between the recent decade (the late 1990 s) and the previous decade in Fig. 4. The raw SAT difference between the hiatus decade and the previous decade in Fig. 4a exhibits a significant difference over the high-latitude land regions of the Northern Hemisphere. It depicts a warming region over Greenland and a cooling region over Siberia. A large-scale cooling region is also found over the Pacific sector. Figure 4b presents the difference of adjusted SAT between two decades. It exhibits a pattern similar to that of the raw SAT in Fig. 4a. Figure 4c depicts the difference in dynamically induced SAT. A cooling region is found over the mid- to high-latitude regions of Eurasia, whereas a strong warming region is found over Greenland in Fig. 4c. Meanwhile, a cooling region appears over the North America, with warming areas occurring to the north of Africa and over the high-latitude regions of the Northern Hemisphere. Comparing Fig. 4c with 4b, a cooling trend is found over the mid- to high-latitude regions of the Eurasia based on the dynamically induced SAT. Additionally, the adjusted SAT exhibits a small scale cooling centre over continental Asia. The strong discrepancy between two periods demonstrated that the source of cooling is the dynamically induced SAT. The dynamical factors always play an important role in atmospheric circulation. One of them or some of them dominated large-scale climate change phenomena^28^,^29^,^30^. Previous results have analysis on the influence of the NAO^9^, PDO^10^,^11^ and AMO^12^,^13^,^14^,^15^ on WTS. Therefore, we calculated the correlative coefficients of NAO (Fig. 5), PDO (Fig. 6) and AMO (Fig. 7) with dynamically induced SAT, separately.

Figure 5a presents a time series of the NAO in the period of 1950–2011 as the Arctic temperature data of GISS is missing before 1950. It illustrates the positive and negative phases of the NAO from 1950 to 2011. The NAO was in its positive phase in the 1950s and became negative in the 1960 s. The strongest positive phase occurred from the 1970 s through 2000. The most pronounced switch from the positive to the negative phase occurred in 2000; the NAO has remained in its negative phase since 2000. Figure 5b shows the distribution of the correlation coefficient between the interdecadal NAO and the dynamically induced SAT. It exhibits a positive pattern over the mid- to high-latitude regions of Eurasia, northwestern North America and nearby the coast of Pacific Ocean. This result illustrates the strong influence of the NAO on the dynamically induced SAT over the Eurasian and North American continents, which is typically observed to exhibit decadal variability. Because the interdecadal NAO time series is negative during the recent hiatus period, the positive correlation coefficient represents the cooling effect on the dynamically induced SAT changes over the mid- to high-latitude areas of Eurasia, northwestern North America and the nearby coast of the Pacific Ocean. The negative correlation coefficient indicates an increase in the dynamically induced SAT over northeastern North America, Africa and the Atlantic. As its important role in dynamically induced SAT, the NAO has also been proposed as a predictor for projecting SAT changes^9^.

Figure 6a is a time series of the PDO in the period of 1950–2011. As shown in the time series of PDO, it did not consistently vary with the NAO over the past 60 years. The PDO began in its negative phase in the 1950 s before entering its positive phase in approximately 1978. The PDO remained in its positive phase until the 2000 s and keeps negative phase until now. It has also been found that the negative phase of the PDO is always associated with more frequent La Nina events; this phenomenon has been independently simulated during the hiatus period in warming scenarios of the 21st century using CCSM4^6^. Figure 6b shows the correlation coefficient between PDO and dynamically induced SAT. The correlation coefficient over northwestern North America and the nearby coast of the Pacific Ocean is positive. The positive correlation coefficient between the PDO and the dynamically induced SAT illustrates that the negative phase of the PDO leads to cooling via dynamically induced SAT changes.

**Figure 8:**
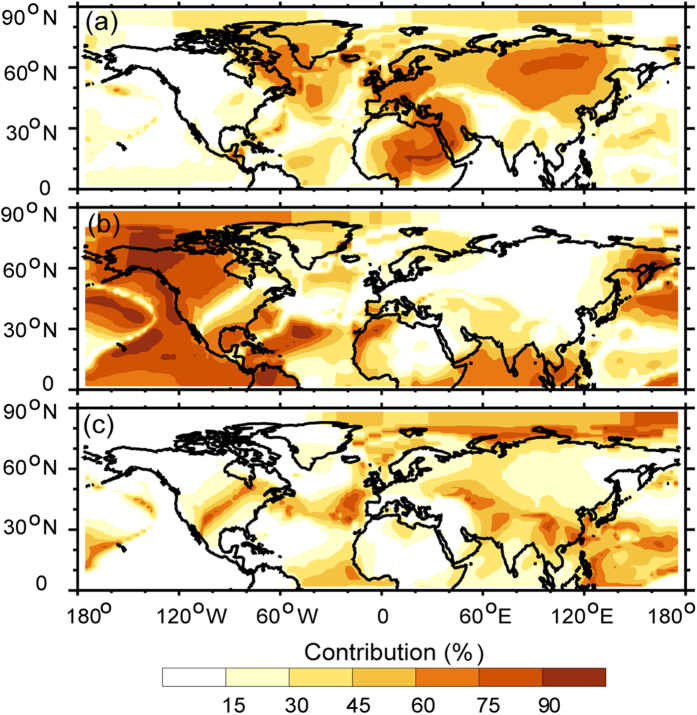
The contribution of the NAO (**a**), PDO (**b**) and AMO (**c**) to the dynamically induced SAT. Maps and plots were made with the Interactive Data Language (IDL) software, IDL Version 8.2, License Number 1251296.

Besides NAO and PDO, AMO is an efficiency index in measuring the dynamical activities^27^. It illustrates that the AMO has been relatively constant since 2000 (Fig. 7a) and exhibits a negative relationship over the mid- to high-latitude regions of Eurasia, western North America and the nearby coast of the Pacific Ocean (Fig. 7b). The relationship between dynamically induced SAT and AMO index is positive over the rest of the Northern Hemisphere, which is represented by the correlation coefficient between AMO and the dynamically induced SAT in Fig. 7b. Figure 7 confirms the role of the AMO in the dynamically induced SAT. The effect of the AMO on the dynamically induced SAT reflects a comprehensive impact over the entire Northern Hemisphere, which was proposed by Wyatt *et al*.^14^,^15^ via the “Stadium wave” theory. In order to summarize the role of NAO, PDO and AMO to the dynamically induced SAT, we exhibited the spatial distribution of contribution in Fig. 8. It illustrates their influences in reducing the dynamically induced SAT during the WTS. Figure 8a illustrates the contribution of the NAO to the dynamically induced SAT over the mid- to high-latitude regions of Eurasia and northern Africa is much more obvious than the other regions and indicates the presence of a relatively weaker centre along the coast of Greenland. The contribution exceeds 60% over the mid- to high-latitude regions of Eurasia and northern Africa, indicating the impact of the NAO on dynamically induced SAT almost focus on these two centres. Figure 8b exhibits the contribution of the PDO to the dynamically induced SAT is significant over western North America and the nearby coast of the Pacific Ocean. Figure 8c depicts the contribution of the AMO to the dynamically induced SAT. The AMO, which represents a comprehensive dynamical factor, does not exhibit any pronounced local maxima.

## Discussion

Although the high correlations of NAO, PDO and AMO with dynamically induced temperature illustrating the stoppage of temperature in the hiatus period are mainly led by dynamic factors, numerical modelling evidence would be informative to address this issue. However, there are a few coupled atmosphere-ocean models can produce the NAO, PDO and AMO simultaneously and the simulated combined effect on SAT has not reported yet. Most simulation studies can only generate a single oscillation mode (i.e., single NAO, PDO or AMO) forced by observed SST^31^,^32^,^33^. Besides, as noted by Wallace *et al*.^21^, the dynamically adjusted method adopted has separated the dynamically induced warming from the observation, exhibiting a uniform spatial pattern of dynamically induced temperature that does not appear in the models. How the NAO, PDO and AMO influence SAT should be an important direction in future study using climate simulations.

Recent studies detailed that the AMO-signal propagation thoughout the Northern Hemisphere via a sequence of atmospheric and lagged oceanic teleconnections, which the authors term the “stadium wave”^14^. The relationship between the NAO and AMO has also been identified by Li *et al*.^9^, and they pointed out that NAO leading AMO by 15–20 years, with a two-way interaction between the NAO and AMO. The magnitude of the NAO forcing of the Atlantic meridional overturning circulation (AMOC)/Atlantic multidecadal oscillation (AMO) and the time delay of the AMOC/AMO feedback are two key parameters of the delayed oscillator^34^. These results suggest, that the hiatus is temporary, and global warming will return when the NAO, PDO and AMO reverse to their positive phases in future. And similar hiatus may occur when the dynamic factors are in negative phase combination next time.

### Data and methods

In this study, we use the observational dataset of monthly SAT from the NASA Goddard Institute for Space Studies (GISS)^21^, which has a spatial resolution of 2° × 2° for 1901–2011, and the historical simulations of 20 CMIP5^25^ climate models for 1901–2005 with the spatial resolution of 0.5° × 0.5°. The CMIP5 models that are listed in Table 1 were introduced in the IPCC AR5. The Representative Concentration Pathways (RCP) 4.5 and RCP 8.5 using the medium-low and highest scenario simulations in CMIP5^25^ provide the data for the RCP simulation during 2005–2011 in Fig. 3. The observational sea level pressure (SLP) data is from the National Oceanic and Atmospheric Administration (NOAA)/Cooperative Institute for Research in Environmental Sciences 20th Century Reanalysis (20CR) version 2. The SLP data have a spatial resolution of 2° × 2° for 1901–2011^35^. The NAO, PDO and AMO indexes were downloaded from Climate Explorer (http://climexp.knmi.nl/).

The dynamical adjustment methodology was first proposed by Wallace *et al*.^21^ to analyse the cause of enhanced warming over the mid- to high-latitude regions of the Northern Hemisphere. The methodology was explicitly introduced by Smoliak *et al*.^36^. The raw SAT in this study is divided into two components based on the dynamical adjustment methodology. The two components are called dynamically induced and adjusted SAT. Wallace *et al*.^21^ noted that the dynamical adjustment is applied to remove the component of the cold season SAT trends over land areas poleward of 20 °N, which are attributable to changing atmospheric circulation patterns.

Dynamical adjustment is based on the regression of sea level pressure to SAT; thus, this methodology removes changes of atmospheric circulation patterns that may be expressed in sea level pressure, which is referred to as dynamically induced SAT variability. After removing the dynamically induced SAT variability, the residual part is associated with radiatively forced factors, such as the build-up of greenhouse gases, stratospheric ozone depletion, volcanic eruptions and aerosol emissions. This component is called the adjusted SAT or radiatively forced SAT variability.

## Additional Information

**How to cite this article**: Guan, X. *et al*. The role of dynamically induced variability in the recent warming trend slowdown over the Northern Hemisphere. *Sci. Rep*. **5**, 12669; doi: 10.1038/srep12669 (2015).

## Figures and Tables

**Table 1:** CMIP5 models examined in this study.

Model name	Modelling centre
BCC-CSM1.1	Beijing Climate Center, China
CanESM2	Canadian Centre for Climate, Canada
CCSM4	National Center for Atmospheric Research, USA
CNRM-CM5	Centre National de Recherches Meteorologiques, France
CSIRO-Mk3.6.0	Commonwealth Scientific and Industrial Research, Australia
GFDL-CM3	Geophysical Fluid Dynamics Laboratory, USA
GFDL-ESM2G	Geophysical Fluid Dynamics Laboratory, USA
GFDL-ESM2M	Geophysical Fluid Dynamics Laboratory, USA
GISS-E2-R	NASA Goddard Institute for Space Studies, USA
HadGEM2-CC	Met Office Hadley Centre, UK
HadGEM2-ES	Met Office Hadley Centre, UK
INM-CM4	Institute for Numerical Mathematics, Russia
IPSL-CM5A-LR	Institute Pierre-Simon Laplace, France
IPSL-CM5A-MR	Institute Pierre-Simon Laplace, France
MIROC-ESM	Japan Agency for Marine-Earth Science and Technology, Japan
MIROC-ESM-CH	Japan Agency for Marine-Earth Science and Technology, Japan
MIROC5	Atmosphere and Ocean Research Institute, Japan
MPI-ESM-LR	Max Planck Institute for Meteorology, Germany
MRI-CGCM3	Meteorological Research Institute, Japan
NorESM1-M	Norwegian Climate Centre, Norway
